# *“He’s probably more Mr. sport than me”* – a qualitative exploration of mothers’ perceptions of fathers’ role in their children’s physical activity

**DOI:** 10.1186/s12887-015-0421-9

**Published:** 2015-08-26

**Authors:** Jesmond Zahra, Simon J. Sebire, Russell Jago

**Affiliations:** Centre for Exercise, Nutrition & Health Sciences, School for Policy Studies, University of Bristol, 8 Priory Road, Bristol, BS8 1TZ, UK

## Abstract

**Background:**

Many children do not meet the recommended levels of physical activity. Parents can influence their children’s physical activity in a number of ways but little research has focused on the impact of fathers. The current study aimed to explore how mothers perceived fathers to influence children’s physical activity.

**Methods:**

Telephone interviews with mothers (*n* = 50) who took part in a large cross sectional physical activity study were conducted. A strategic sampling method was applied to ensure varying deprivation levels and child physical activity. Interviews were based on children’s physical activity and screen viewing behaviours and patterns. A total of 37 interviews included information on fathers and were used for the current study. Deductive content analysis was used to analyse the interviews.

**Results:**

Mothers suggested that fathers are directly involved in their child’s physical activity though co-participation, whilst additionally playing an important role in encouraging and facilitating physical activity. The results suggest some variation in how mothers and fathers are involved in children’s physical activity behaviours. Father availability seems to play a key role in the amount of physical activity involvement.

**Conclusions:**

Fathers play a key role in children’s physical activity choices and behaviours and can influence children in a variety of ways. Parents tend to share in the physical activity related tasks of their children but father availability seems to be a factor in their amount of involvement. Health professionals aiming to improve child physical activity may benefit from developing interventions that target both children and fathers.

## Background

Low physical activity (PA) levels are associated with increased risk of coronary heart disease, hypertension, type 2 diabetes and certain cancers [1]. An inactive lifestyle is the fourth leading risk factor for global mortality, accounting for approximately six percent of global deaths per year [2]. It is estimated that approximately 20–35 % of cardiovascular diseases could be prevented if individuals adopted more active lifestyles [3]. The cost of physical inactivity within the UK is estimated at approximately £900 million per year putting substantial strain on the National Health Service [4].

The UK Department of Health [5] recommends that adults engage in least 30 min of PA on at least 5 days of the week. Within England only 39 % of men and 29 % of women report meeting this recommendation [6]. The UK Department of Health [5] guidelines for young people aged 5 to 18 years recommend at least an hour of PA per day; currently only 32 % of boys and 24 % of girls meet these PA levels [6]. Several studies have suggested that PA patterns in childhood track into adulthood [7, 8], emphasising a need to tackle childhood inactivity.

A number of studies have provided evidence of the ways in which parents influence their children’s PA [9–13]. A recent cross sectional study highlighted that parents influence PA of children aged 9 to 10 years through a variety of mechanisms including modelling and encouraging behaviours [12]. A further quantitative study, utilising accelerometery, identified four factors related to higher PA in children aged 9 years; lower age of mother, higher parental participation, parental facilitation, and higher PA of mothers and of fathers. A number of these factors could be targeted in interventions aiming to improve child PA levels [13]. Despite the use of an objective measure of PA the study offers little insight into exactly how parents participate in or facilitate children’s PA.

A recent qualitative study explored the perceptions of PA behaviours in working parents of children below the age of 18 by using focus groups. Participants reported a variety of barriers to the PA of themselves and their children, including family responsibilities, lack of support and scheduling constraints. Parents highlighted a number of ways in which they facilitate their child’s PA behaviours such as role modelling behaviour or prioritising PA. Whilst these findings offer an insight into parental influence on child PA, the generalizability of the findings are somewhat limited. The participants had children of a variety of ages, were recruited via a university emailing list and were highly educated and affluent [14].

With these studies in mind, PA interventions targeting parents could have the potential to improve both child and adult PA levels [15]. Health research involving children and parents has tended to focus on mothers and thus largely neglected the role of fathers [16, 17]. Fathers tend to be more difficult to recruit into parent–child studies and this could partially explain the tendency to recruit higher proportions of mothers in these types of studies [18, 19]. This recruitment bias is highlighted by recent literature reviews examining the participation rates in PA interventions which show an underrepresentation of male participants in PA research [20, 21]. Furthermore, research exploring parenting programmes has reported a need to adapt services to improve the uptake of fathers [22, 23]. For example Sure Start, a UK government funded initiative which provides an opportunity for parents to be active with their child during programme sessions, has acknowledged the need to tailor their services to improve father uptake [22].

Fathers are becoming more involved in child-care and home environments [24] but there is little research on whether they may impact upon children’s PA choices and behaviours. The relative lack of male participants in intervention trials makes it difficult to draw conclusions on the role fathers may play in children’s PA. An exception is the Australian Healthy Dads Healthy Kids study [25–27] which specifically targeted fathers and children. The Healthy Dads Healthy Kids study showed that an intervention targeting fathers and children can improve PA levels; fathers and children in the intervention arm of the study engaged in significantly greater amounts of PA than those in the control arm. Specifically, after six months of the study, the mean difference in steps per day between groups was 2327 for children and 3546 for fathers. This study highlights the benefit of targeting fathers and the importance of developing an understanding of fathers’ role in children’s PA behaviours and the wider family context [25–27].

The majority of studies exploring PA behaviours have tended to be quantitative, allowing for few conclusions to be drawn regarding the mechanisms or motivations behind these behaviours [28]. Within the realm of family research specifically, qualitative studies may offer an insight into how PA behaviours and patterns may be developed and maintained during childhood and the relative influence of members within the family unit.

Thomas et al. recognise the need for more qualitative studies describing qualitative research in PA as the “new kid on the block”[28]. As it stands there is insufficient qualitative work and even fewer studies that have specifically explored the relationship between father and child activity. The current study aims to address this gap in research by exploring the variety of ways fathers’ impact on child PA patterns and behaviours. Specifically we sought to investigate a) how fathers facilitate children’s PA; b) the ways fathers encourage children’s PA; and c) whether their availability has an effect on their influence. Previous literature has suggested that mothers’ perceptions of the paternal role predict father involvement more accurately than fathers’ own perceptions, suggesting that there is a lack of connection between fathers’ perceptions of their parenting role and their actual behaviour [29]. In order to gain a less biased view on these questions, we interviewed mothers. Additionally, through exploring mothers’ views we hoped to explore the variations between mothers and fathers in relation to child PA. This study will add to the current literature exploring parent and child PA relationships whilst providing further insight into how PA behaviours in young children are established, influenced and maintained throughout childhood and the relative impact fathers have on the PA within this age group.

## Methods

The current study utilised data collected in the B-Proact1v Study; a large cross-sectional project that aimed to explore parental and child PA behaviours. Study methods have been published elsewhere [30, 31]. Briefly however, between Jan 2012 and May 2013, 250 primary schools in and around Bristol were invited to participate in the study; a total of 57 schools took part in data collection. B-Proact1v recruited year one pupils (5–6 years of age), and at least one parent/carer. In total 410 triads (mother, father and child), 212 father-child and 645 mother-child dyads provided accelerometer data. Participating dyads (parent/ s-child) were asked to complete a questionnaire and wear an accelerometer (GT3X+) for five days. Those participants whose child provided at least 3 valid days of accelerometer wear time (500 min to allow estimation of PA level), a valid postcode and address to allow for calculation of socioeconomic position (SEP) and consent to be contacted were included in the sampling frame for interviewing. Ethical approval was granted by a University of Bristol ethics committee and informed consent was collected from participants prior to data collection [30, 31].

A random stratified sampling method was used to ensure varying degrees of SEP, based upon postal code Index of Multiple Deprivation (IMD) scores, and a range of active to inactive children (based on time spent performing moderate to vigorous physical activity - MVPA). This procedure was conducted by using thirds of MVPA and IMD to calculate low, medium and high MVPA/SEP categories, creating a three by three matrix (9 groups). In total 274 participants were randomly selected to be contacted for interviews; 30 % of participants were male. Batches of participants were selected five times during the study to allow data collection to be staggered. Following selection, participants were invited to participate initially by letter and then by phone. Participants were given a £10 high street shopping voucher for their time. A process of saturation was used to determine the number of interviews. Specifically, regular debrief meetings were held after initial reading of transcripts and interviewing ceased when no new information was forthcoming.

### Semi structured interviews

Telephone interviews were selected as the method of data collection as they provide a cost effective way of collecting information and allow more flexibility for the participant and research team [32]. Interviews were recorded with an Olympus DS-3400 digital recorder. An initial set of interview questions were developed by the research team through exploring literature on parenting and PA and identifying gaps in qualitative and quantitative knowledge. These included questions relating to a variety of topics, for example, influences of friends on child PA, parental perceptions of their child’s activity, screen viewing behaviours of children and alternatives to screen viewing. During the interview process questions were developed iteratively from interview feedback and team meetings at numerous points during the interview process. This process resulted in a number of additional questions, one of which related to how other family members, such as the child’s siblings or father influences PA choices and behaviours.

Over a three month period, 50 interviews were completed with mothers; three were completed with fathers. Father interviews were not included in the analysis to reduce bias. However, to support findings from mothers, a brief overview is included at the end of the results section. For the purpose of this paper the 37 interviews in which mothers referred to father impact or role are utilised. The average interview duration was approximately 26 min. Of those included in the analysis the average age of mothers was 38.8 (SD. 5.7) years of age, 11 % had one child, 62 % two children and the remaining 27 % had more than two children. The sample included parents from different ethnicities but was predominantly White British (89 %). 19 % of participants were unemployed/full time parents, 62 % worked part time and 19 % worked full time. Table 1 displays the representation of participants in the matrix.

**Table 1:** Spread of participants

	High SEP	Medium SEP	Low SEP
Low MVPA	3	3	3
Medium MVPA	9	5	4
High MVPA	4	3	3

### Analysis

Interviews were transcribed verbatim and anonymised before being entered to QSR NVivo 10 (QSR International, Warrington UK) to facilitate analysis. A deductive content analysis method was used to analyse the complete interview data as outlined by Elo et al. [33]. The process involved three main steps; preparation, organisation and reporting. Firstly, the research team repeatedly read through the interviews to become immersed in the data. During this phase multiple themes were identified from the literature and previous research conducted by the research team. An unconstrained matrix was used to allow for the creation of additional categories for data that did not fit the original matrix of analysis. A category that emerged from the data when examining family member’s roles in child PA was the influence of fathers. Mind maps were developed that specifically related to the role of fathers, this aided in the identification of categories. The team identified four main categories from the data relating to fathers’ roles in child PA; role modelling, facilitation and encouragement, co-physical activity and weekend involvement/availability. These were discussed in depth by the research team to ensure that they appropriately captured all the data. During the organisation phase units of analysis were coded into these themes. Dual coding was performed to ensure that participant responses were categorised correctly and the results were agreed upon by members of the team. Regular peer meetings were scheduled throughout the analysis process to enable the discussion of emerging themes and to confirm agreement between members of the research team. Any disagreements that occurred during coding were discussed with additional members of the research team in order to ensure consensus.

## Results

Participant responses were coded into a number of main themes; a) ways in which fathers influence and encourage PA; b) how they role model positive activity behaviour; c) their weekend involvement and d) direct influence on children’s PA. Once responses had been coded, further exploration was performed to probe variations in views on the basis of child gender and household IMD scores. Below each quotation the child’s gender and MVPA category is reported, along with the parental SEP category as calculated from their postal code. Initial analysis of the data suggested minimal variations in responses between SEP categories, as such SEP was not considered further. Once the analyses had been completed, illustrative quotes for each theme were extracted and discussed among the authors until the final quotes were selected.

### Get on our bikes and go - fathers encouragement and facilitation

Parents facilitate and influence PA in a number of different ways, including taking children to sports clubs, encouraging outings to the park and enrolling their children into PA lessons. Participants suggested these activity-related tasks tend to be equally shared between parenting partners. The below quotes highlight some of the ways in which fathers facilitate in their children’s PA behaviours without necessarily being involved in the activity themselves.

*“Their dad takes them um at the weekends up to the football club, and there’s a playground up there, sort of play around and also they go and watch football and stuff, so they walk to and from there.” Male/Low SEP/Low MVPA*

*“Yeah he’s really good I’d say, like he’s, I’m much better at kind of just carrying out tasks. You know like if they’ve got swimming lessons, I’m happy to take them, but [father name] is like really kind of on it that they need to get the lessons, do you know what I mean and he will kind of go out of his way to make sure that is happening, whereas I can just let it drift where it just all gets too much …” Female/Medium SEP/Medium MVPA*

The quotes suggest that fathers are an important means of encouragement for children’s PA behaviours. Furthermore, fathers provide logistic support for their children to be active through providing an environment that enables PA, in this case a park/playground, or alternatively by transporting them to a club in which they can be active. The quotes are also suggestive of the different roles mothers and fathers fulfil. Mothers imply that fathers are more involved in encouragement and instigation of activity whereas their role involves the practicalities of getting children to clubs or activities. Some mothers seemed more involved in the academic development of the child whereas the father is interested in PA.

*“He’s into sport, he’s all about sport, so I’m the one, he’s probably more Mr. sport than me, I will sit down with [participating child] and do the spellings and the readings for example, and he will say let's go in the garden…” Female/Medium SEP/Medium MVPA*

The quote above suggests that for this father, PA is important and he prompts his child to be physically active. Additionally, the quote provides an example of how fathers may instigate co-participation in PA with their children. Interestingly, when the participating child was female and mothers were prompted about father involvement some mothers responded in relation to father-son activities as opposed to father-daughter.

*“…while I take [participating child] to tennis he takes [younger brother] to Rugby, he will take [younger brother] straight to the park and I will take [participating child] to ballet, so we sort of divide our time a little bit…” Female/High SEP/Medium MVPA*

*“I mean if we take [participating child] anywhere then my husband’s at work, then often I’d do that but he takes equal sort of share in terms of weekends and things like that. Takes [younger brother] to football and things like that, so yeah there’s definitely an equal share….” Female/High SEP/Medium MVPA*

These responses highlight that fathers may be more inclined to facilitate with their sons PA rather than their daughters. It should be acknowledged that these quotes related to families with multiple children of both genders. Fathers’ role and/or impact on girls PA maybe more apparent in families in which there are just girls.

### *He likes to do what dad does* – fathers as active role models

Role modelling can be describe as carrying out a behaviour that can be emulated by others, in this case by children. The interviews indicated that mothers perceived that fathers may be an important PA role model for their children.

*“Yeah, I mean my husband’s very into fitness… and so my son sees him out running and riding his bike and swimming and everything and my husband would take both of them out on their bikes” Male/High SEP/Low MVPA*

*“… he will go out for a run quite a lot and he will go for bike rides quite a lot and so yeah, the kids will think it’s really normal to say where’s Daddy, oh he’s gone for a run. So yeah they would be very influenced by him and he’s … yeah, he’s very active” Male/High SEP/High MVPA*

The responses from mothers imply that children are aware of when their father is being active and this may have some role in their choice of PA behaviours and the degree to which PA is a “*normal*” adult behaviour. Furthermore, mothers commonly referred to the fathers’ (and less often their own) personal interest in fitness as a source of potential influence:

*“… because his dad does it so he likes to do what dad does….He does … he's quite … he's quite high up in his Judo and Jiu jitsu so the boys have always done that since they were little. All of them” Male/Low SEP/High MVPA (quote in relation to judo club)*

Some mothers went further and described their desire as parents, who both exercise, to model an active environment which encourages their child’s PA through links with health and enjoyment.

*“me and my husband both exercise regularly, we kind of want to get him into that pattern, where he sees it as being healthy and good for you and, and do something he enjoys” Male/Low SEP/Medium MVPA*

Others suggest that role modelling may just be a by-product of parental tendencies to be physical active or enjoy specific activities.

*“…my husband’s quite into football, but, [participating child] is the one who chose it, … but it’s probably influenced a bit by the family sort of football orientation, or my husband” Male/High SEP/Medium MVPA*

Whist a large number of mothers discussed fathers’ role modelling behaviours, a large proportion of these discussions arose when the participating child was male. There was some evidence that fathers may role model behaviours for their daughters, but the type of activity fathers enjoy doing may not be appropriate for children of this age group.

*“She wants to go to gym, she’s too young obviously but she would like to go to gym with him [father], she likes running, riding, on her bike, yeah.” Female child/Middle SEP/Medium MVPA*

### *All of us together –* physical activity co-participation

Co-participating in PA involves parents being physically active with their child or children, for example taking them to the park and actively running around with them, or playing a sport with them. Mothers reported that fathers often co-participate in a number of different activities ranging from structured activities such as golf, to unstructured such as play fighting.

*“Yeah he goes off to the driving range, his dad takes him and he goes off on the driving range and he gets fifty balls and he really enjoys that.” Male/Middle SEP/Medium MVPA*

*“…my husband is a great walker, he loves the great outdoors and he would always take the children for long walks either in the woods or somewhere further afield” Female/Low SEP/Medium MVPA*

*“Well actually all of us together, me my husband and [Participating child], he likes to play fight with his father. They do a lot of play fight and me and my husband we always liked kickboxing, never had the chance to do it, so we thought oh we’ll ask would you like to go and try” Male/Low SEP/Medium MVPA*

Participants suggested that fathers’ co-participation in PA may be different to their own. For example the quote below suggests that the father maybe more involved in activities such as impromptu trips to the park.

*“he'd say, ‘Right, let’s run over the playground’, we live next to a playground. So, he’ll do something physical with them then.” Female/High SEP/Low MVPA*

The second quote further emphasises these differences, the mother is involved more with the organisational side of child PA whereas the father is involved in the active travel to and from the club. In the case below, the father takes along all his children thereby increasing not just one child’s PA.

*“Yeah I do the admin and the initial finding out about, ‘Oh she … they can do this club’, and then, you know, I, I sort of arrange the clubs, but then like she's got her athletics club on a Monday and quite often my husband will cycle her there and then pick her up on the bike and take, you know, the other two with him” Female/High SEP/Low MVPA*

Mothers’ views suggested that co-participation may be a function of the parents’ perceived competence in the specific activity.

*“It’s … that would be equally shared. He very much does the swimming more so then I do, because he’s a better swimmer really” Female/Low SEP/High MVPA*

Further differences in parental co-participation were identified by one mother who compared her passive behaviour to fathers’ active participation.

*“So if you go to a park with them they are at your lead kind of thing following them around, whereas if they get a bit older like [participating child] and [Sibling]’s age, they are off so you can sit and natter with the mums. Whereas I think if you watch the dad’s, the children that are running about are with their dads… probably be kicking a ball around” Male/High SEP/Low MVPA*

### *Daddy’s around at the weekend:* fathers’ presence and involvement

Mothers were consistent in their reference to the importance of father availability, mainly relating to their work patterns in the week and at weekends. The findings did not vary according to child gender or socioeconomic position. Furthermore, the quotes suggest that father availability plays a role in their involvement. The quotes below emphasise that when fathers are free from other commitments, such as work, they are keen to be active with their child/children.

*“Daddy takes him swimming, because daddy’s around at the weekend.” Male/Middle SEP /Medium MVPA*

*“she would rather walk and if my husband is available in the morning he(father) takes her, they walk to school” Female/Low SEP/Medium MVPA*

*“During the week they spend more time with me. So in the weekends he tends to like go out with them and have a walk with them or play football with them or you know, all stuff like that, and I’m here cooking or sitting and relaxing.” Male/Low SEP/Medium MVPA*

Some mothers referred to the complexity of juggling family life around work patterns and the potential influence this may have on PA.

*“… my husband works full time, I work his days off, basically, and I work sort of twelve, thirteen hour shifts, so my husband has a big role in their bringing up as well and … yes, and I think, you know, he encourages it as well, probably more so than me. Probably my husband would encourage more physical activity with them than probably me, so …” Male/High SEP/Medium MVPA*

*“Yeah, well my husband works a lot so I would say, well he does do a lot on the weekend with them, so I would say yeah it’s equal, because in the weekend he is on and I’m off”. Male/Low SEP/Medium MVPA*

The above quotations imply fathers are more available at the weekend and therefore may have more potential to influence children’s PA behaviours at this time.

### Father’s interviews

The three interviews conducted with fathers support the data above and a number of illustrative quotes can be viewed in Table 2. Consistent with the mother reports, fathers reported instigating and performing a variety of structured and unstructured activities with their children. Furthermore, the interviews suggested that fathers are keen to encourage, facilitate and co-participate in PA with children. Moreover, fathers are likely to respond to children’s requests to be physically active. Similarly to the information provided by mothers, work patterns were suggested to be a factor when considering the amount of PA carried out. Fathers did not suggest they were role models for PA; this may be due to the limited number of interviews performed or a lack of awareness regarding the impact they have on their children’s PA choices and behaviours.

**Table 2:** Data from father interviews

Father interview 1	Father interview 2	Father interview 3
Male/high SEP/medium MVPA	Female/medium SEP/low MVPA	Male/low SEP/low MVPA
*Availability/Co-participation*	*Encouragement*	*Encouragement*
*“In terms of like getting home of an evening or over a weekend, he said “dad would go and play” I don’t know “cricket or something out the front”, there is a little plastic cricket set and it will be him instigating that.”*	*“if she’s watching something, and she’s watched enough, then we will say right that’s enough now, let’s go out, let’s go out in the garden and play instead.”*	*“I do say he should be more active and let’s go and do this and more often than not he will, yeah, he’ll stop what he was doing and he’ll come with me.”*
*Facilitation*	*Co-Participation*	*Co-Participation*
*“The thought of dumping him somewhere and leaving him, he probably wouldn’t take to, and that would probably put him off, but if he knew you were going to be there the first few times, he would be happy.”*	*yeah we go out and we go out and play, run around together, so um, or we’ll go to the park and they ride their scooters and run around in there.”*	*“Erm, what I do is I erm, we’ve got quite a big back garden and I use the ball, and we either kick it for you know, ground catch, play catch or we’ve got a basketball net here, so erm, yes I’ll just say let’s go out in the garden and err, do something.”*

## Discussion

The current study used interviews to explore mothers’ perception of fathers’ involvement in the PA of children aged 5–6 years. The findings suggest that fathers play a key role in promoting children’s PA and influencing their choices and behaviours. The data from this study indicates that fathers could be an important figure for children of this age group and have an impact on PA through a number of different mechanisms. These mechanisms include supporting PA by facilitating, actively travelling, role modelling and co-participating in sport and activities. As outlined earlier, studies have previously explored the impact that parents may have on children PA, however there is limited research conducted with parents of children this age, and even fewer have focused specifically on the role of fathers. Therefore little is known on the extent to which fathers’ impact on their children’s PA, thus the current study adds to the literature.

Parents act as motivators, encouragers and facilitators of PA by supporting children to carry out activities. Mothers suggested that fathers facilitate and encourage PA in a variety of ways, ranging from verbally encouraging to co-participating in activity. Interestingly, responses from mothers varied depending on the gender of the participating child. For example, when discussing father involvement, mothers often referred to father-son activity despite the interview being based on their daughters PA. This suggests that the responsibilities of each parent may vary depending on the gender of the child. This idea is supported by quantitative evidence suggesting gender specific associations between parent and child PA [34]. This is also commensurate with previous research suggesting that mothers are more likely than fathers to support their daughter’s activity by enrolling them in physical activities and taking them to these events [9]. The current results, along with previous literature, suggests that fathers could be more motivated or inclined to participate in activity with their son compared with their daughter. Although the reasoning behind this variation is relatively unexplored, it could be a function of a shared enthusiasm for similar sports or activities.

Role modelling can promote observational learning and act as a prompt for previously learned behaviours [35]. Research has shown that having a PA role model is associated with lower risk of being overweight [36] and positive role modelling has been shown to be an important predictor of children’s PA [11]. The present results suggest that fathers may be important role models for their children. Mothers’ responses imply they understand the importance of having an active role model and that their children are aware of when their father’s active. Furthermore, there is some suggestion from mothers that this may help foster a “normalisation” of PA from the child’s perspective. However, it is important to acknowledge that other studies have suggested parental role modelling does not independently result in increased child PA, but rather parent/child PA relationships or associations are likely to be developed through more complex mechanisms and result from multiple factors [37, 38]. With this in mind, role modelling may be an important factor in encouraging positive health behaviours for children, but additional support, encouragement or facilitation maybe required in order to directly impact on health behaviours.

Mothers in the present study reported that fathers co-participate in activities with their children. These range from unstructured play in the park to structured activities such as golf or tennis. Evidence has highlighted the importance of parental social support, indicating an increase in PA when parents carryout activities with their children [39]. Furthermore, social support is an important predictor of children’s involvement and interest in PA and joint play/activity is likely to have a lasting impact [40]. The data from the current study highlights that fathers are jointly active with their children and often encourage activity. This supports findings by Davison and colleagues who found fathers were more likely than mothers to use their own behaviour to encourage activity [9].

Although fathers are involved in PA activities with their children, it is difficult to determine when these activities occur. The results from the current study suggest that fathers’ availability is greater at the weekend and this may have an impact on child PA behaviours and levels. The results are in line with previous research that found parents of 10–11 year olds are more physically active with their children during the weekend [41]. Increased availability is likely to be a function of the work patterns of fathers; which may in part explain the variation between weekday and weekend father involvement in child activity. Interestingly, mothers portray the weekend as their time off from child-care, implying that the father will often take the lead role in regards to weekend activities.

PA interventions targeting children have had limited success [42, 43]. Family-based interventions have tended to recruit more mothers in comparison to fathers, meaning that there is a lack of research exploring the effectiveness of family-based interventions involving fathers [44]. Fathers’ parenting style has been shown to have greater influence on pre-school children’s weight status in comparison to the mothers’ [45] and having an overweight father is associated with an increased risk for childhood obesity [46]. These studies highlight the importance of including fathers in family-based research whilst additionally emphasising that fathers play an influential role in children’s behaviours and choices. Furthermore, the recent Healthy Dad’s Healthy Kid’s study highlights the potential impact fathers can have on their children’s PA levels [25–27].

### Strengths and limitations

The collection of quantitative data prior to interviews enabled us to interview individuals from a variety of socioeconomic positions with children ranging from active to inactive. Furthermore, the large sample size enabled us to gain an in-depth understanding of the impact fathers have on children’s PA, and illustrated the wide range of factors that may mediate this impact. Though this study provides valuable information on the impact fathers have on children’s PA, our interpretations are based on mothers’ opinions which limits our understanding of the motivations driving these behaviours. Although our findings are supported by the data from three father interviews, it is likely that further interviews with additional fathers may have provided a more insightful understanding of their impact on child PA.

Some of the participating mothers failed to provide information on the influence of fathers. This may have been due to their family situation, for example single parent families, or due to a lack of prompting on behalf of the interviewers. As our questions developed as the interviews progressed, questions relating to fathers were not in our initial interview schedule but were introduced in response to information provided by mothers during initial interviews. Additionally, our findings are restricted to the ways in which fathers may influence child PA and offer little insight into fathers’ influence on the intensity of their child’s PA, therefore we cannot ascertain whether fathers are having a direct or indirect impact on children’s MVPA. Furthermore, although we found that mothers’ perceived fathers’ to facilitate, encourage and co-participate in their child’s activity related behaviours, we cannot presume these are the only ways in which fathers support their child’s activity. We would need to interview additional fathers to fully understand the extent of their involvement and influence. Although our study was open to all parents and children attending the schools we recruited a predominantly white British sample which limits the generalizability of the findings.

Furthermore, while our sampling process aimed to ensure we interviewed parents from a variety of SEPs, mothers from low SEPs are slightly under represented in the current study, as demonstrated in Table 1, and this should be considered when interpreting the findings. A further limitation arises from focusing interviews on only one child which may have resulted in the exclusion of important information regarding fathers’ involvement with older or younger siblings. By focusing on a specific age group (i.e. 5–6 year olds) any variation in PA behaviours or parental impact as children age is missed. It would be beneficial to understand the age range in which parents have the greatest impact on children’s PA and at what age peer influence becomes of more importance. Although it was beyond the scope of this study, it would be interesting to explore whether specific father behaviours, for example role modelling, are more or less influential than other behaviours such as co-participating in PA. Despite these limitations the study provides a good insight into the variety of ways fathers can influence children’s activity patterns and behaviours. The large sample size and range of participants that took part is a further strength of the study.

## Conclusions

The data presented here suggest that targeting fathers in PA interventions may be an effective strategy for improving children’s PA behaviour. Our findings could be used to design interventions that seek to encourage parents to be more active with their children or modify already recognised behaviours such as modelling PA, encouraging and co-participating in PA. By highlighting theses type of behaviour to fathers during interventions, researchers may find it easier to modify and encourage future father-child PA interactions. Furthermore, the data from the study highlights time periods that fathers are available to be active with their children. Specifically, the weekend may be an appropriate time to encourage PA behaviours between fathers and children. Further, as fathers are a difficult population to recruit into parenting interventions [47] our findings suggest that researchers aiming to recruit fathers should consider scheduling interventions at the weekend to improve recruitment rates and desirability.

In summary the data indicates that fathers are able to influence the amount and type of PA children participate. With this in mind health professionals and other agencies working with parents may do well to encourage fathers to adopt these types of methods to encourage PA in children from a relatively young age.

## Abbreviations

PA: Physical activity
MVPA: Moderate to vigorous physical activity
WHO: World health organisation
IMD: Index of multiple deprivation
SEP: Socio-economic position
